# Distance from human settlements favors wild‐type appearance of feral cats (*Felis catus*) in Mediterranean woodland

**DOI:** 10.1002/ece3.10261

**Published:** 2023-07-03

**Authors:** Shahar Dubiner, Itai Namir, Ron Chen, Eran Levin

**Affiliations:** ^1^ School of Zoology Tel Aviv University Tel Aviv Israel; ^2^ Hamaarag—Israel's National Nature Assessment Program, The Steinhardt Museum of Natural History Tel Aviv University Tel Aviv Israel

**Keywords:** camouflage, cats, coloration, crypsis, domestication, *Felis catus*, rural

## Abstract

Camouflage is a common trait enabling animals to avoid detection by predators and prey. Patterns such as spots and stripes are convergent across carnivore families, including felids, and are hypothesized to have adaptive value through camouflage. House cats (*Felis catus*) were domesticated thousands of years ago, but despite artificial selection for a wide variety of coat colors, the wild‐type pattern of tabby cats is very common. We aimed to determine whether this pattern grants an advantage over other morphs in natural environments. We collected cat images taken with camera traps in natural areas near and far from 38 rural settlements in Israel, to compare the habitat use by feral cats of different colors. We tested the effect of proximity to villages and habitat vegetation (normalized difference vegetation index, NDVI) on the probability of space use by the tabby morph compared to the others. NDVI had a positive effect on site use in both morphs, but non‐tabby cats had a 2.1 higher probability of using the near sites than the far sites, independent of NDVI. The wild‐type tabby cats' probability of site use were equally likely to be unaffected by proximity, or have an interaction of proximity with NDVI whereby the far transects are used with increasing probability in sites of denser vegetation. We hypothesize that the camouflage of tabby cats, more than other colors and patterns, confers an advantage in roaming the woodland habitats for which this pattern evolved. This has both theoretical implications as rare empirical evidence of the adaptive value of fur coloration, and practical implications on managing the ecological impact of feral cats worldwide.

## Graphical abstract

We collected house cat images taken with camera traps in natural areas near and far from villages, to compare the habitat use by feral cats of different pelage colors. The space use by cats with non‐wild‐type coloration is lower in the far cameras compared to the near ones, whereas wild‐type tabby appearance does not change with proximity. We ttribute this to the tabbies' camouflage in the woodland region, as supported by a positive correlation of vegetation and far site usage.

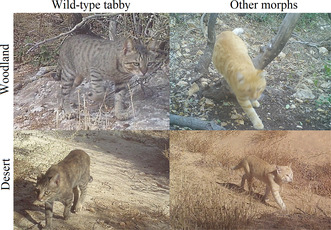

## 1 INTRODUCTION

Camouflage has been suggested as the most important evolutionary force explaining color adaptation in mammals (Cuthill, 2019). Most carnivore color patterns are associated with specific environmental variables (Ortolani, 1999), suggesting that the animal's physical environment is an important selective force driving the evolution of these patterns. Felids are among the most specialized carnivores, whose primary hunting strategy is stalking prey until the prey is close enough to be captured by a pounce or quick rush; hunts are more successful as the distance for an attack is reduced (Murray et al., 1995). Camouflage may allow felids to remain undetected for as long as possible, thereby increasing the chance of a successful attack (Cuthill, 2019). In the smaller cat species, camouflage also provides essential protection from predation by other carnivores (Palomares & Caro, 1999). House cats (*Felis catus*) are the most widely distributed carnivore in the world and are known to be commensal with humans (Driscoll et al., 2009; Grimm, 2009). Because feral house cat densities are not limited by territoriality (Liberg & Sandell, 2000), the abundant supply of food provided by humans, directly or indirectly, facilitates high cat densities near and around human settlements (Finkler et al., 2011; Grimm, 2009; Mirmovitch, 1995). Feral cats in rural areas use natural environments for hunting and mating (Bengsen et al., 2012; Fitzgerald & Karl, 1986; Liberg, 1980; McGregor et al., 2015), and are often found to be roaming further than 1 km from the core of their home range (Fitzgerald & Karl, 1986; Liberg, 1980). This carries dire implications for small local species, which suffer predation from both owned and feral domestic cats (Bonnaud et al., 2011; Hardman et al., 2016; Mella‐Méndez et al., 2022). Being among the 100 worst invasive species in the world (Lowe et al., 2000), there is a need to study of factors such as pelage coloration that may influence their spatial patterns of hunting in the wild.

Due to their domestic history, house cat coloration has undergone different selective pressure than wild felids, and house cats express a wide range of colors and patterns. In contrast to other domestic animals, artificial selection on cats occurs mainly on color patterns and less on other traits (Kaelin & Barsh, 2013). The genetic basis of cat color morphs is well understood (Kaelin & Barsh, 2013), and according to current knowledge, they are not directly linked to behavior. A recent survey of cat owners suggests that coat colors may be associated with aggressive behaviors, or that tabbies are bolder and more active than other color morphs (González‐Ramírez & Landero‐Hernández, 2022); however, if these differences exist, they are relatively minor (Stelow et al., 2016).

The variety in fur color of feral house cats provides an opportunity to examine how color differences affect habitat use by a small carnivore and examine hypotheses that relate habitat use to coloration. We assume there is an adaptive advantage in natural habitats for cats that are similar in color and pattern to the grayish tabby appearance of the wildcat (*Felis silvestris*). Previous studies on cat coloration suggested that coat patterns are likely to be adaptive for camouflage rather than communication or physiological reasons (Mella‐Méndez et al., 2022; Randall et al., 1987; Todd, 1977). Other studies suggest that felids using more “closed” environments like woodlands and arboreal locomotion are more likely to have complex patterns than those using open habitats and terrestrial locomotion (Allen et al., 2011). Moreover, the quantity of prey hunted by domestic cats has been shown to correlate with the cat's coat color (Mella‐Méndez et al., 2022). Hunting by domestic cats can have a substantial and diverse impact on local fauna (Bonnaud et al., 2011; Brickner‐Braun et al., 2007; Ferreira et al., 2011; Hardman et al., 2016; McGregor et al., 2015; Mella‐Méndez et al., 2022), which calls for research on the intrinsic and extrinsic factors determining the patterns of cats' habitat use. Our main hypothesis was that cat coloration affected their resource use, specifically that the tabby morph, which is similar to *F. silvestris* and presumably better camouflaged in woodlands, would be able to be more active in these natural areas. We therefore predicted the probability of finding wild‐like tabby cats relative to other color morphs to be higher away from human settlements. We also predicted the difference to be related to habitat type, since denser vegetation provides better camouflage.

## 2 METHODS

We used camera traps (PC900, RECONYX, USA, and BTC 6HDPX Dark Ops) deployed as part of Israel's National Terrestrial Biodiversity Monitoring Program run by Hamaarag—Israel's National Nature Assessment Program (Sorek & Shapira, 2018), to record habitat utilization by cats in the natural areas around each of the 38 villages, for 10 consecutive days and nights on five different years between 2013 and 2022 (which were divided into five biennial campaigns). Cameras operated continuously during the 10 days. We positioned a transect of nine cameras near to each village (typically 100 m from the village edge into the natural area, but varying according to local topography and conditions), and another transect of nine cameras far from each village (500–2000 m from the village edge into the natural area; Appendix 1). Cameras were positioned in 85 m intervals, 45 cm above ground, attached to a tree trunk or a metal pole, and facing the trail at 60°. The cameras were activated by motion (high sensitivity of 0.3 s of movement to trigger the camera) and were programmed to shoot five photos in 0.5 s intervals when triggered. For each camera trap that captured a cat photo, we determined whether cats were tabby or some alternative coloration (e.g., ginger, white, black, piebald, calico) as a binomial variable. When pelage color pattern could not be discerned with certainty, the image was discarded. To have the most conservative estimate of site use by the cats, we assigned presence/absence to each of the transects (near or far), for each of which the sampling effort was nine cameras times 10‐days per campaign. We obtained the normalized difference vegetation index (NDVI) for each camera's location from Sorek and Shapira (2018). We ran two generalized linear mixed models, one with presence/absence of tabby cats as response factor and one with presence/absence of the other morphs. We chose the predictors for each model according to AICc using a model selection approach, with initial fixed factors being NDVI, proximity (near vs. far), village population, their various interactions, and a null model. For all models, site location (village name) and year (campaign number) were added as random factors. We ensured no overdispersion (*ĉ* not greater than 1 for all models) using the “check_overdispersion” function in the R package “performance” (Lüdecke et al., 2021).

## 3 RESULTS

Across the five 10‐day campaigns, a total of 191 surveys in 36 of the 38 sites captured discernable images of house cats. Of these, wild‐like tabby cats were observed in 58 surveys of the far transects and 57 of the near ones, compared to 33 cats of other coat patterns in the far transects and 59 in the near ones (Appendix 1).

**FIGURE 1:**
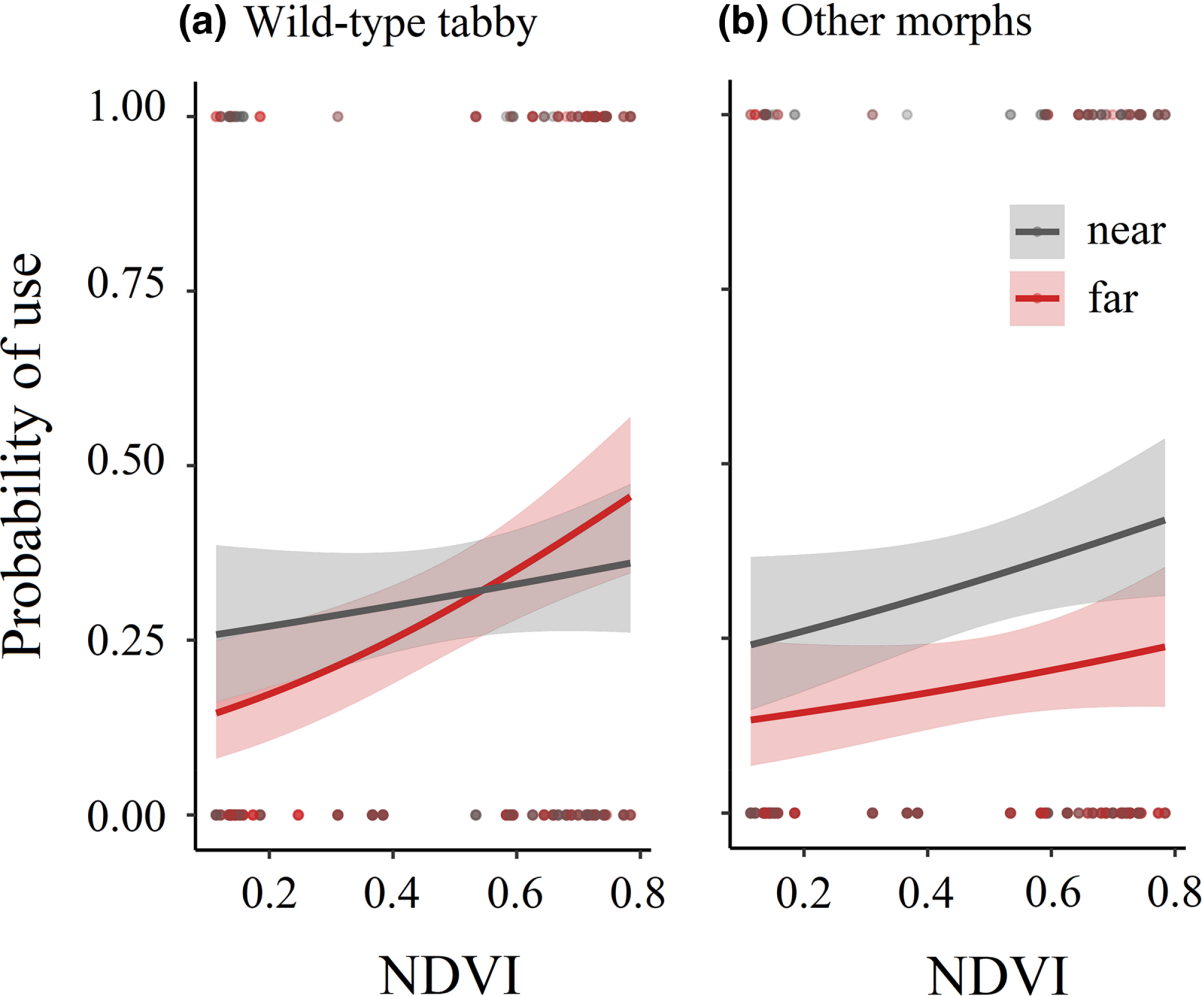
The probabilities of site use for tabby cats and other morphs as a function of proximity to the nearest villages and NDVI. For tabby cats, probability of use in the best models was either not predicted by proximity, or increased with NDVI more strongly in the far transects than the near ones (a). For other color patterns, the transects far from the villages had lower probability of use than the near ones, independent of the effects of NDVI (b).

The two best models for site use by tabby cats included NDVI alone (ΔAICc = 0, weight = 0.403), as well as an interaction model of proximity and NDVI (ΔAICc = 0.92, weight = 0.255; Table 1a). Both models were >8 times more likely than the null model. According to the interaction model, for tabby cats, the probability of site use was 1.9 times higher for an NDVI increase of one in the near transects, compared to 2.9 times higher for an NDVI increase of one in the far transects (Figure 1a). The two best models for site use by non‐tabby cats included proximity and NDVI (ΔAICc = 0, weight = 0.401), as well as proximity, NDVI, and population (ΔAICc = 0.54, weight = 0.307; Table 1b). According to the first model, the probability of site use by non‐tabby cats was 2.8 times higher for an NDVI increase of one, and (independently of NDVI) 2.1 times higher in the near transects compared to the far ones (Figure 1b). The model including population was very similar, but predicted the site use probability to be 1.04 times higher per 1000 people. Both these models were >100 times more likely than the null model, and >75 times more likely than any model that did not include proximity to human settlements as an explaining variable.

**TABLE 1:** Model selection tables used to select the best models for (a) tabby cats and (b) other morphs.

Intercept	Proximity	NDVI	NDVI × Proximity	Population	NDVI × population	Population × proximity	NDVI × population × proximity	Df	logLik	AICc	Delta	Weight
(a) Tabby cats												
−1.8400		1.7530						4	−211.348	430.8	0.00	0.403
−1.3710	+	0.8668	+					6	−209.745	431.7	0.92	0.255
−1.8280	+	1.7520						5	−211.343	432.9	2.05	0.145
−1.3230	+	0.8377	+	−0.00003				7	−209.683	433.7	2.87	0.096
−1.7860	+	1.7290		−0.00003				6	−211.293	434.8	4.01	0.054
−0.9994								3	−214.952	436.0	5.16	0.030
−0.9823	+							4	−214.943	438.0	7.19	0.011
−1.3190	+	0.9477	+	0.00	−0.0001	+	+	10	−209.528	439.7	8.86	0.005
(b) Other morphs												
−2.246	+	1.227						5	−189.988	390.1	0.00	0.401
−2.442	+	1.378		0.0001				6	−189.220	390.7	0.54	0.307
−2.169	+	1.080	+					6	−189.961	392.2	2.02	0.146
−2.375	+	1.252	+	0.0001				7	−189.201	392.7	2.58	0.110
−2.296	+	1.623	+	0.0001	−0.0006	+	+	10	−187.303	395.3	5.10	0.031
−1.735		1.184						4	−195.597	399.3	9.16	0.004
−1.852	+							4	−197.906	403.9	13.77	0.000
−1.352								3	−203.551	413.2	23.02	0.000

*Note*: All models included the random factors “campaign” and “location”.

## 4 DISCUSSION

**FIGURE 2:**
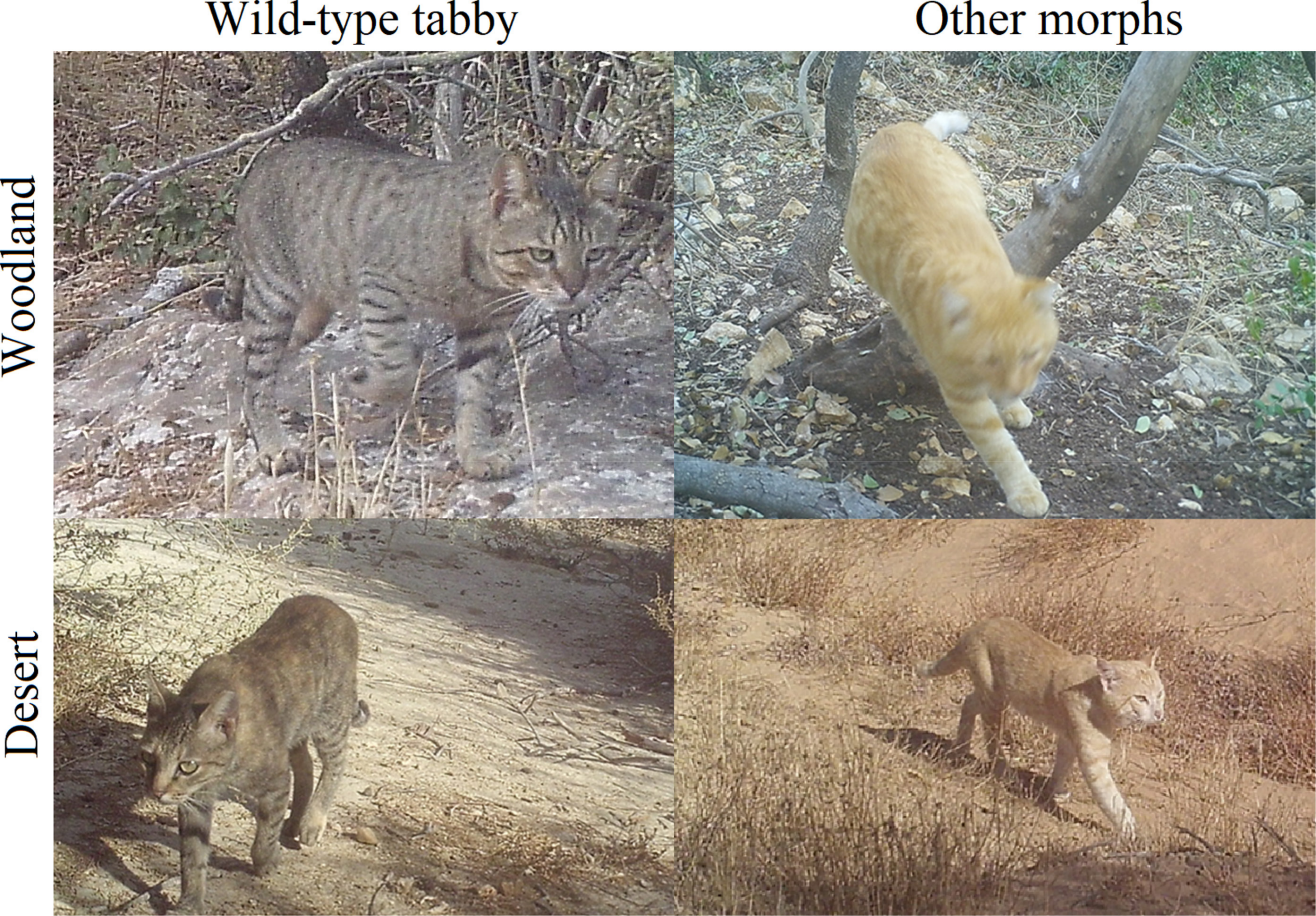
The benefit of camouflage, granted by the wild‐type tabby coloration (left column) over other color patterns (right column) in Mediterranean woodland areas (top row), apparently loses its advantage in desert habitats (bottom row).

Due to its high frequency, tabby coloration in cats has been suggested to have an adaptive value, but the selective driving mechanisms remain unclear (Dards & Robinson, 1983; Todd, 1977; Wagner & Wolsan, 1987). There was a general tendency of cats to be present in more vegetated areas (higher NDVI) across all models, perhaps since they would be more exposed in open habitats, or due to lower population densities in the desert. In line with our predictions, the best models indicated that cats with the various artificially selected color patterns have lower site use probability farther from human settlements, whereas wild‐type tabby cats are less influenced by distance from humans. The two (equally likely) models for wild‐type tabby cats were either no effect of proximity at all (only NDVI), or an interaction whereby increasing NDVI counteracts this effect (i.e., more vegetation increases the probability to use the far sites). This can be explained by the adaptive value of the tabby pattern's camouflage in dense vegetation (Figure 2). Many animals select habitats in which they, or their offspring, are visually cryptic to potential predators (Caro, 2014; Cuthill, 2019). A pelage color that makes the cat difficult to distinguish from the background by pattern matching mechanism (crypsis, Endler, 1981) could be an important factor for avoiding being killed by larger carnivores. Adult house cats and their kittens are reported to be preyed on by other carnivores, like the Egyptian mongoose, red fox, and golden jackal (Ferreira et al., 2011; Appendix 2) all observed in high densities within both regions in Israel. Ferreira et al. (2011) suggested that the presence and abundance of competing predators mediate the differences in the presence, abundance, and movements of cats in natural areas. The high density of predators in the Mediterranean regions of Israel (mainly due to anthropogenic activity) might cause a similar pressure on cats inhabiting these habitats, giving an advantage to a more cryptic pelage color in natural environments. Given that across species of wild felids, irregular and intricate patterns are an adaptation for wooded habitats (Allen et al., 2011), we suggest that cat pelage coloration might affect the habitat use of cats in Mediterranean woodland and similar habitats. Konecny (1983), who found differences in fur color between two cat populations in the Galápagos, offered a non‐adaptive explanation to the dominance of gray tabby cats in the more isolated population (over 70 km away from the nearest village), attributing the difference to founder effects and lack of recruitment. However, in our study this explanation is ruled out by the relatively short distances, the large number of populations examined, and correlation to habitat strengthening the conclusion of camouflage. Although many of the behavioral characteristics of wildcats were preserved in house cats during their domestication by humans (mainly the hunting skills and the urge to hunt), color was one of the characteristics that were selectively and artificially modified (Kaelin & Barsh, 2013; Montague et al., 2014). This character might be selectively balanced toward the wild‐type morph when cats are back in their wild environments. It is possible that this trend is due to a learning process, rather than selection, whereby cats with conspicuous pelage have more incentive to keep close to humans if rates of predator encounters and unsuccessful hunting are higher in the wild.

Another possible alternative explanation for the correlation in our data is that due to the difficulty in distinguishing between house cats (*F. catus*) and wildcats (*F. silvestris*; Daniels et al., 2001), we might have erroneously labeled wildcats as tabby house cats. In parts of their distribution, wildcats are known to hybridize with domestic cats extensively, and then higher ratios of the tabby morph in the feral cat population can be expected (Beaumont et al., 2001; Pierpaoli et al., 2003; Randi, 2008; Yamaguchi et al., 2004). Based on extensive data collected in Israel, Mendelssohn and Yom‐Tov (1999) suggested that in the Mediterranean part of Israel, wildcats are all hybridized with house cats. Hybridized cats also use both natural and human‐supplied food sources (Biró et al., 2005; Germain et al., 2008). The high density of settlements along the Mediterranean region of Israel left very small areas of natural environments, limiting any separation between populations of house cats and wildcats—but also not allowing for very large wildcat populations in the first place. The short distances, less that a cat can walk in a night, also weaken (but do not rule out) the possibility that cats nearer human settlements with higher level of ownership simply reflect the artificial color preferences of cat owners (e.g., Farnworth et al., 2018). Lastly, tabbies could potentially carry genes related to a tendency for roaming and hunting behavior, but to the best of our knowledge this kind of linkage between color morph and behavior are weak or nonexistent (see Stelow et al., 2016). Based on our data, which represents an exceptional sampling effort gathered over many years and locations, we conclude that the natural coloration of spots and stripes has an adaptive value rooted in its camouflage in the woodland habitat in which it evolved.

Our results provide new evidence of the adaptive value of camouflage in mammals. Besides its theoretical significance, this may carry some practical implications: feral cats preying on smaller animals in natural and semi‐natural areas pose a threat, often a serious one, to the local fauna, including endangered species (Bonnaud et al., 2011; Hardman et al., 2016; Mella‐Méndez et al., 2022). Therefore, understanding what affects their hunting success (McGregor et al., 2015; Mella‐Méndez et al., 2022) and occupancy of the surrounding habitat (Ferreira et al., 2011) is of value to conservation.

## AUTHOR CONTRIBUTIONS

**Shahar Dubiner:** Data curation (lead); formal analysis (lead); investigation (lead); visualization (lead); writing – original draft (equal); writing – review and editing (equal). **Itai Namir:** Data curation (equal); formal analysis (supporting); investigation (supporting); writing – review and editing (supporting). **Ron Chen:** Data curation (supporting); project administration (equal); resources (lead); writing – review and editing (supporting). **Eran Levin:** Conceptualization (lead); project administration (equal); supervision (lead); writing – original draft (equal); writing – review and editing (equal).

## FUNDING INFORMATION

This study was partially funded by the Azrieli Graduate Studies Fellowship.

## CONFLICT OF INTEREST STATEMENT

The authors declare no conflict of interest.

## Data Availability

All the relevant data are provided in the text and Appendix 1.

## FULL SURVEY RESULTS

**Table:**

Tabby	Non‐tabby	Campaign	Location	Proximity	Habitat	Population	NDVI
0	1	T0	Aderet	Near	Wood	850	0.5833
0	0	T0	Aderet	Far	Wood	850	0.5833
1	0	T0	Beer_milka	Near	Desert	150	0.1575
0	0	T0	Beer_milka	Far	Desert	150	0.1575
0	0	T0	Beit_oren	Far	Wood	500	0.659
1	1	T0	Beit_oren	Near	Wood	500	0.659
0	0	T0	Beit_yatir	Far	Desert transition zone	600	0.3668
0	1	T0	Beit_yatir	Near	Desert transition zone	600	0.3668
1	0	T0	Bislach	Far	Desert	Na	0.143
0	0	T0	Bislach	Near	Desert	Na	0.143
1	0	T0	Ein_yaakov	Far	Wood	1200	0.7423
1	1	T0	Ein_yaakov	Near	Wood	1200	0.7423
0	0	T0	Ein_yahav	Far	Desert	800	0.1465
1	0	T0	Ein_yahav	Near	Desert	800	0.1465
0	0	T0	Ezuz	Far	Desert	100	0.1371
1	0	T0	Ezuz	Near	Desert	100	0.1371
1	0	T0	Gamla	Far	Wood	550	0.7273
1	0	T0	Gamla	Near	Wood	550	0.7273
1	1	T0	Gvat_yearim	Far	Wood	1400	0.6443
1	1	T0	Givat_yearim	Near	Wood	1400	0.6443
0	0	T0	Givat_yeshayahu	Far	Wood	800	0.6805
0	1	T0	Givat_yeshayahu	Near	Wood	800	0.6805
0	1	T0	Goren	Far	Wood	600	0.7838
0	0	T0	Goren	Near	Wood	600	0.7838
0	0	T0	Har_amasa	Far	Desert transition zone	200	0.3837
0	0	T0	Har_amasa	Near	Desert transition zone	200	0.3837
1	1	T0	Iftach	Far	Wood	600	0.6884
0	0	T0	Iftach	Near	Wood	600	0.6884
0	0	T0	Karei_deshe	Far	Wood	Na	0.6994
1	0	T0	Karei_deshe	Near	Wood	Na	0.6994
1	1	T0	Kerem_maharal	Far	Wood	800	0.7219
1	1	T0	Kerem_maharal	Near	Wood	800	0.7219
1	0	T0	Kfar_shamai	Far	Wood	550	0.667
0	1	T0	Kfar_shamai	Near	Wood	550	0.667
1	0	T0	Kisalon	Far	Wood	450	0.5339
0	1	T0	Kisalon	Near	Wood	450	0.5339
0	0	T0	Lahav	Far	Desert transition zone	650	0.5939
1	0	T0	Lahav	Near	Desert transition zone	650	0.5939
1	1	T0	Lehavim	Far	Desert transition zone	6500	0.3108
0	1	T0	Lehavim	Near	Desert transition zone	6500	0.3108
1	1	T0	Lotan	Far	Desert	150	0.121
0	0	T0	Lotan	Near	Desert	150	0.121
0	1	T0	Margaliot	Far	Wood	400	0.5897
0	1	T0	Margaliot	Near	Wood	400	0.5897
1	0	T0	Merhav_am	Far	Desert	500	0.185
0	0	T0	Merhav_am	Near	Desert	500	0.185
1	0	T0	Mirsham	Far	Desert transition zone	Na	0.6258
1	0	T0	Mirsham	Near	Desert transition zone	Na	0.6258
0	0	T0	Natur	Far	Wood	900	0.7151
0	0	T0	Natur	Near	Wood	900	0.7151
1	0	T0	Nehusha	Far	Wood	1500	0.7269
0	0	T0	Nehusha	Near	Wood	1500	0.7269
1	0	T0	Nir_etzion	Far	Wood	950	0.7126
1	1	T0	Nir_etzion	Near	Wood	950	0.7126
1	0	T0	Ofer	Far	Wood	650	0.7731
0	0	T0	Ofer	Near	Wood	650	0.7731
1	0	T0	Paran	Far	Desert	500	0.114
0	1	T0	Paran	Near	Desert	500	0.114
1	0	T0	Sde_boker	Far	Desert	450	0.1345
1	0	T0	Sde_boker	Near	Desert	450	0.1345
0	0	T0	Sekher	Far	Desert	Na	0.2468
0	1	T0	Sekher	Near	Desert	Na	0.2468
0	0	T0	Shaal	Far	Wood	300	0.7405
1	0	T0	Shaal	Near	Wood	300	0.7405
0	0	T0	Shunra	Far	Desert	Na	0.1735
0	1	T0	Shunra	Near	Desert	Na	0.1735
1	0	T0	Yagur	Far	Wood	1650	0.7449
1	0	T0	Yagur	Near	Wood	1650	0.7449
1	0	T0	Yavneel	Far	Wood	4450	0.7152
1	0	T0	Yavneel	Near	Wood	4450	0.7152
0	0	T0	Yeruham	Far	Desert	10,200	0.1384
1	0	T0	Yeruham	Near	Desert	10,200	0.1384
0	0	T0	Yotvata	Far	Desert	700	0.1522
1	0	T0	Yotvata	Near	Desert	700	0.1522
0	0	T0	Zofar	Far	Desert	450	0.1368
0	0	T0	Zofar	Near	Desert	450	0.1368
0	0	T1	Aderet	Near	Wood	850	0.5833
0	0	T1	Aderet	Far	Wood	850	0.5833
0	0	T1	Beer_milka	Near	Desert	150	0.1575
0	0	T1	Beer_milka	Far	Desert	150	0.1575
0	0	T1	Beit_oren	Far	Wood	500	0.659
0	0	T1	Beit_oren	Near	Wood	500	0.659
0	0	T1	Beit_yatir	Far	Desert transition zone	600	0.3668
0	0	T1	Beit_yatir	Near	Desert transition zone	600	0.3668
0	0	T1	Bislach	Far	Desert	Na	0.143
0	1	T1	Bislach	Near	Desert	Na	0.143
0	1	T1	Ein_yaakov	Far	Wood	1200	0.7423
0	0	T1	Ein_yaakov	Near	Wood	1200	0.7423
0	0	T1	Ein_yahav	Far	Desert	800	0.1465
0	1	T1	Ein_yahav	Near	Desert	800	0.1465
1	0	T1	Ezuz	Far	Desert	100	0.1371
0	0	T1	Ezuz	Near	Desert	100	0.1371
1	0	T1	Gamla	Far	Wood	550	0.7273
0	0	T1	Gamla	Near	Wood	550	0.7273
0	0	T1	Givat_yearim	Far	Wood	1400	0.6443
0	0	T1	Givat_yearim	Near	Wood	1400	0.6443
0	0	T1	Givat_yeshayahu	Far	Wood	800	0.6805
0	0	T1	Givat_yeshayahu	Near	Wood	800	0.6805
0	0	T1	Goren	Far	Wood	600	0.7838
1	0	T1	Goren	Near	Wood	600	0.7838
0	0	T1	Har_amasa	Far	Desert transition zone	200	0.3837
0	0	T1	Har_amasa	Near	Desert transition zone	200	0.3837
0	0	T1	Iftach	Far	Wood	600	0.6884
0	0	T1	Iftach	Near	Wood	600	0.6884
0	0	T1	Karei_deshe	Far	Wood	Na	0.6994
1	0	T1	Karei_deshe	Near	Wood	Na	0.6994
0	0	T1	Kerem_maharal	Far	Wood	800	0.7219
0	0	T1	Kerem_maharal	Near	Wood	800	0.7219
0	0	T1	Kfar_shamai	Far	Wood	550	0.667
0	0	T1	Kfar_shamai	Near	Wood	550	0.667
0	1	T1	Kisalon	Far	Wood	450	0.5339
0	0	T1	Kisalon	Near	Wood	450	0.5339
0	0	T1	Lahav	Far	Desert transition zone	650	0.5939
0	0	T1	Lahav	Near	Desert transition zone	650	0.5939
0	0	T1	Lehavim	Far	Desert transition zone	6500	0.3108
0	0	T1	Lehavim	Near	Desert transition zone	6500	0.3108
0	0	T1	Lotan	Far	Desert	150	0.121
0	0	T1	Lotan	Near	Desert	150	0.121
0	0	T1	Margaliot	Far	Wood	400	0.5897
0	1	T1	Margaliot	Near	Wood	400	0.5897
1	0	T1	Merhav_am	Far	Desert	500	0.185
0	1	T1	Merhav_am	Near	Desert	500	0.185
0	0	T1	Mirsham	Far	Desert transition zone	Na	0.6258
0	0	T1	Mirsham	Near	Desert transition zone	Na	0.6258
0	0	T1	Natur	Far	Wood	900	0.7151
0	1	T1	Natur	Near	Wood	900	0.7151
0	0	T1	Nehusha	Far	Wood	1500	0.7269
0	1	T1	Nehusha	Near	Wood	1500	0.7269
0	0	T1	Nir_etzion	Far	Wood	950	0.7126
1	0	T1	Nir_etzion	Near	Wood	950	0.7126
0	0	T1	Ofer	Far	Wood	650	0.7731
0	0	T1	Ofer	Near	Wood	650	0.7731
0	0	T1	Paran	Far	Desert	500	0.114
0	0	T1	Paran	Near	Desert	500	0.114
0	0	T1	Sde_boker	Far	Desert	450	0.1345
0	1	T1	Sde_boker	Near	Desert	450	0.1345
0	0	T1	Sekher	Far	Desert	Na	0.2468
0	0	T1	Sekher	Near	Desert	Na	0.2468
1	0	T1	Shaal	Far	Wood	300	0.7405
1	0	T1	Shaal	Near	Wood	300	0.7405
0	0	T1	Shunra	Far	Desert	Na	0.1735
0	0	T1	Shunra	Near	Desert	Na	0.1735
1	0	T1	Yagur	Far	Wood	1650	0.7449
0	0	T1	Yagur	Near	Wood	1650	0.7449
0	1	T1	Yavneel	Far	Wood	4450	0.7152
0	1	T1	Yavneel	Near	Wood	4450	0.7152
0	0	T1	Yeruham	Far	Desert	10,200	0.1384
0	0	T1	Yeruham	Near	Desert	10,200	0.1384
0	0	T1	Yotvata	Far	Desert	700	0.1522
1	0	T1	Yotvata	Near	Desert	700	0.1522
0	1	T1	Zofar	Far	Desert	450	0.1368
0	1	T1	Zofar	Near	Desert	450	0.1368
0	0	T2	Aderet	Near	Wood	850	0.5833
0	0	T2	Aderet	Far	Wood	850	0.5833
0	0	T2	Beer_milka	Near	Desert	150	0.1575
0	0	T2	Beer_milka	Far	Desert	150	0.1575
0	0	T2	Beit_oren	Far	Wood	500	0.659
0	0	T2	Beit_oren	Near	Wood	500	0.659
0	1	T2	Beit_yatir	Far	Desert transition zone	600	0.3668
0	0	T2	Beit_yatir	Near	Desert transition zone	600	0.3668
1	0	T2	Bislach	Far	Desert	Na	0.143
0	0	T2	Bislach	Near	Desert	Na	0.143
1	0	T2	Ein_yaakov	Far	Wood	1200	0.7423
0	0	T2	Ein_yaakov	Near	Wood	1200	0.7423
0	0	T2	Ein_yahav	Far	Desert	800	0.1465
0	0	T2	Ein_yahav	Near	Desert	800	0.1465
0	0	T2	Ezuz	Far	Desert	100	0.1371
0	0	T2	Ezuz	Near	Desert	100	0.1371
1	0	T2	Gamla	Far	Wood	550	0.7273
1	0	T2	Gamla	Near	Wood	550	0.7273
0	0	T2	Givat_yearim	Far	Wood	1400	0.6443
0	0	T2	Givat_yearim	Near	Wood	1400	0.6443
0	0	T2	Givat_yeshayahu	Far	Wood	800	0.6805
0	1	T2	Givat_yeshayahu	Near	Wood	800	0.6805
0	0	T2	Goren	Far	Wood	600	0.7838
1	0	T2	Goren	Near	Wood	600	0.7838
0	1	T2	Har_amasa	Far	Desert transition zone	200	0.3837
0	0	T2	Har_amasa	Near	Desert transition zone	200	0.3837
0	0	T2	Iftach	Far	Wood	600	0.6884
0	0	T2	Iftach	Near	Wood	600	0.6884
0	1	T2	Karei_deshe	Far	Wood	Na	0.6994
1	0	T2	Karei_deshe	Near	Wood	Na	0.6994
0	1	T2	Kerem_maharal	Far	Wood	800	0.7219
0	0	T2	Kerem_maharal	Near	Wood	800	0.7219
0	0	T2	Kfar_shamai	Far	Wood	550	0.667
0	0	T2	Kfar_shamai	Near	Wood	550	0.667
1	0	T2	Kisalon	Far	Wood	450	0.5339
0	0	T2	Kisalon	Near	Wood	450	0.5339
0	0	T2	Lahav	Far	Desert transition zone	650	0.5939
1	0	T2	Lahav	Near	Desert transition zone	650	0.5939
0	1	T2	Lehavim	Far	Desert transition zone	6500	0.3108
0	1	T2	Lehavim	Near	Desert transition zone	6500	0.3108
1	0	T2	Lotan	Far	Desert	150	0.121
1	1	T2	Lotan	Near	Desert	150	0.121
0	0	T2	Margaliot	Far	Wood	400	0.5897
0	0	T2	Margaliot	Near	Wood	400	0.5897
1	0	T2	Merhav_am	Far	Desert	500	0.185
0	0	T2	Merhav_am	Near	Desert	500	0.185
0	0	T2	Mirsham	Far	Desert transition zone	Na	0.6258
0	0	T2	Mirsham	Near	Desert transition zone	Na	0.6258
0	0	T2	Natur	Far	Wood	900	0.7151
0	1	T2	Natur	Near	Wood	900	0.7151
0	0	T2	Nehusha	Far	Wood	1500	0.7269
0	0	T2	Nehusha	Near	Wood	1500	0.7269
0	0	T2	Nir_etzion	Far	Wood	950	0.7126
1	1	T2	Nir_etzion	Near	Wood	950	0.7126
0	0	T2	Ofer	Far	Wood	650	0.7731
0	0	T2	Ofer	Near	Wood	650	0.7731
0	1	T2	Paran	Far	Desert	500	0.114
0	1	T2	Paran	Near	Desert	500	0.114
0	0	T2	Sde_boker	Far	Desert	450	0.1345
0	0	T2	Sde_boker	Near	Desert	450	0.1345
0	1	T2	Sekher	Far	Desert	Na	0.2468
0	0	T2	Sekher	Near	Desert	Na	0.2468
0	0	T2	Shaal	Far	Wood	300	0.7405
0	1	T2	Shaal	Near	Wood	300	0.7405
0	0	T2	Shunra	Far	Desert	Na	0.1735
0	0	T2	Shunra	Near	Desert	Na	0.1735
0	0	T2	Yagur	Far	Wood	1650	0.7449
1	1	T2	Yagur	Near	Wood	1650	0.7449
0	1	T2	Yavneel	Far	Wood	4450	0.7152
0	1	T2	Yavneel	Near	Wood	4450	0.7152
0	0	T2	Yeruham	Far	Desert	10,200	0.1384
0	0	T2	Yeruham	Near	Desert	10,200	0.1384
0	1	T2	Yotvata	Far	Desert	700	0.1522
0	0	T2	Yotvata	Near	Desert	700	0.1522
0	1	T2	Zofar	Far	Desert	450	0.1368
1	0	T2	Zofar	Near	Desert	450	0.1368
0	0	T3	Aderet	Near	Wood	850	0.5833
0	0	T3	Aderet	Far	Wood	850	0.5833
1	1	T3	Beer_milka	Near	Desert	150	0.1575
0	1	T3	Beer_milka	Far	Desert	150	0.1575
0	1	T3	Beit_oren	Far	Wood	500	0.659
0	1	T3	Beit_oren	Near	Wood	500	0.659
0	0	T3	Beit_yatir	Far	Desert transition zone	600	0.3668
0	0	T3	Beit_yatir	Near	Desert transition zone	600	0.3668
0	0	T3	Bislach	Far	Desert	Na	0.143
0	0	T3	Bislach	Near	Desert	Na	0.143
1	1	T3	Ein_yaakov	Far	Wood	1200	0.7423
1	0	T3	Ein_yaakov	Near	Wood	1200	0.7423
0	0	T3	Ein_yahav	Far	Desert	800	0.1465
1	0	T3	Ein_yahav	Near	Desert	800	0.1465
0	1	T3	Ezuz	Far	Desert	100	0.1371
1	1	T3	Ezuz	Near	Desert	100	0.1371
1	0	T3	Gamla	Far	Wood	550	0.7273
1	0	T3	Gamla	Near	Wood	550	0.7273
0	1	T3	Givat_yearim	Far	Wood	1400	0.6443
0	0	T3	Givat_yearim	Near	Wood	1400	0.6443
0	0	T3	Givat_yeshayahu	Far	Wood	800	0.6805
0	0	T3	Givat_yeshayahu	Near	Wood	800	0.6805
1	0	T3	Goren	Far	Wood	600	0.7838
0	0	T3	Goren	Near	Wood	600	0.7838
0	0	T3	Har_amasa	Far	Desert transition zone	200	0.3837
0	1	T3	Har_amasa	Near	Desert transition zone	200	0.3837
1	0	T3	Iftach	Far	Wood	600	0.6884
0	1	T3	Iftach	Near	Wood	600	0.6884
0	0	T3	Karei_deshe	Far	Wood	Na	0.6994
0	0	T3	Karei_deshe	Near	Wood	Na	0.6994
0	0	T3	Kerem_maharal	Far	Wood	800	0.7219
1	0	T3	Kerem_maharal	Near	Wood	800	0.7219
1	0	T3	Kfar_shamai	Far	Wood	550	0.667
0	1	T3	Kfar_shamai	Near	Wood	550	0.667
1	0	T3	Kisalon	Far	Wood	450	0.5339
0	1	T3	Kisalon	Near	Wood	450	0.5339
0	0	T3	Lahav	Far	Desert transition zone	650	0.5939
1	0	T3	Lahav	Near	Desert transition zone	650	0.5939
0	0	T3	Lehavim	Far	Desert transition zone	6500	0.3108
1	0	T3	Lehavim	Near	Desert transition zone	6500	0.3108
0	0	T3	Lotan	Far	Desert	150	0.121
1	0	T3	Lotan	Near	Desert	150	0.121
0	0	T3	Margaliot	Far	Wood	400	0.5897
0	1	T3	Margaliot	Near	Wood	400	0.5897
0	0	T3	Merhav_am	Far	Desert	500	0.185
0	0	T3	Merhav_am	Near	Desert	500	0.185
1	1	T3	Mirsham	Far	Desert transition zone	Na	0.6258
1	0	T3	Mirsham	Near	Desert transition zone	Na	0.6258
0	0	T3	Natur	Far	Wood	900	0.7151
0	1	T3	Natur	Near	Wood	900	0.7151
0	0	T3	Nehusha	Far	Wood	1500	0.7269
0	0	T3	Nehusha	Near	Wood	1500	0.7269
0	1	T3	Nir_etzion	Far	Wood	950	0.7126
0	0	T3	Nir_etzion	Near	Wood	950	0.7126
0	0	T3	Ofer	Far	Wood	650	0.7731
0	1	T3	Ofer	Near	Wood	650	0.7731
0	1	T3	Paran	Far	Desert	500	0.114
0	1	T3	Paran	Near	Desert	500	0.114
0	0	T3	Sde_boker	Far	Desert	450	0.1345
1	0	T3	Sde_boker	Near	Desert	450	0.1345
0	0	T3	Sekher	Far	Desert	Na	0.2468
0	0	T3	Sekher	Near	Desert	Na	0.2468
1	0	T3	Shaal	Far	Wood	300	0.7405
0	0	T3	Shaal	Near	Wood	300	0.7405
0	0	T3	Shunra	Far	Desert	Na	0.1735
0	0	T3	Shunra	Near	Desert	Na	0.1735
1	0	T3	Yagur	Far	Wood	1650	0.7449
1	0	T3	Yagur	Near	Wood	1650	0.7449
0	0	T3	Yavneel	Far	Wood	4450	0.7152
0	0	T3	Yavneel	Near	Wood	4450	0.7152
0	1	T3	Yeruham	Far	Desert	10,200	0.1384
0	1	T3	Yeruham	Near	Desert	10,200	0.1384
0	0	T3	Yotvata	Far	Desert	700	0.1522
0	1	T3	Yotvata	Near	Desert	700	0.1522
0	0	T3	Zofar	Far	Desert	450	0.1368
0	1	T3	Zofar	Near	Desert	450	0.1368
1	0	T4	Aderet	Near	Wood	850	0.5833
0	0	T4	Aderet	Far	Wood	850	0.5833
1	0	T4	Beer_milka	Near	Desert	150	0.1575
0	1	T4	Beer_milka	Far	Desert	150	0.1575
0	0	T4	Beit_oren	Far	Wood	500	0.659
0	0	T4	Beit_oren	Near	Wood	500	0.659
0	0	T4	Beit_yatir	Far	Desert transition zone	600	0.3668
0	0	T4	Beit_yatir	Near	Desert transition zone	600	0.3668
0	0	T4	Bislach	Far	Desert	Na	0.143
0	0	T4	Bislach	Near	Desert	Na	0.143
1	0	T4	Ein_yaakov	Far	Wood	1200	0.7423
0	1	T4	Ein_yaakov	Near	Wood	1200	0.7423
0	1	T4	Ein_yahav	Far	Desert	800	0.1465
1	1	T4	Ein_yahav	Near	Desert	800	0.1465
0	0	T4	Ezuz	Far	Desert	100	0.1371
0	0	T4	Ezuz	Near	Desert	100	0.1371
1	0	T4	Gamla	Far	Wood	550	0.7273
0	0	T4	Gamla	Near	Wood	550	0.7273
0	0	T4	Givat_yearim	Far	Wood	1400	0.6443
1	1	T4	Givat_yearim	Near	Wood	1400	0.6443
1	0	T4	Givat_yeshayahu	Far	Wood	800	0.6805
0	1	T4	Givat_yeshayahu	Near	Wood	800	0.6805
1	0	T4	Goren	Far	Wood	600	0.7838
1	0	T4	Goren	Near	Wood	600	0.7838
0	0	T4	Har_amasa	Far	Desert transition zone	200	0.3837
0	1	T4	Har_amasa	Near	Desert transition zone	200	0.3837
0	0	T4	Iftach	Far	Wood	600	0.6884
1	1	T4	Iftach	Near	Wood	600	0.6884
1	1	T4	Karei_deshe	Far	Wood	Na	0.6994
0	1	T4	Karei_deshe	Near	Wood	Na	0.6994
1	1	T4	Kerem_maharal	Far	Wood	800	0.7219
1	0	T4	Kerem_maharal	Near	Wood	800	0.7219
1	0	T4	Kfar_shamai	Far	Wood	550	0.667
1	0	T4	Kfar_shamai	Near	Wood	550	0.667
1	0	T4	Kisalon	Far	Wood	450	0.5339
1	0	T4	Kisalon	Near	Wood	450	0.5339
0	1	T4	Lahav	Far	Desert transition zone	650	0.5939
0	1	T4	Lahav	Near	Desert transition zone	650	0.5939
0	0	T4	Lehavim	Far	Desert transition zone	6500	0.3108
0	0	T4	Lehavim	Near	Desert transition zone	6500	0.3108
0	0	T4	Lotan	Far	Desert	150	0.121
0	1	T4	Lotan	Near	Desert	150	0.121
1	0	T4	margaliot	Far	Wood	400	0.5897
1	0	T4	margaliot	Near	Wood	400	0.5897
0	0	T4	merhav_am	Far	Desert	500	0.185
0	1	T4	merhav_am	Near	Desert	500	0.185
1	0	T4	Mirsham	Far	Desert transition zone	Na	0.6258
0	0	T4	Mirsham	Near	Desert transition zone	Na	0.6258
1	0	T4	Natur	Far	Wood	900	0.7151
0	0	T4	Natur	Near	Wood	900	0.7151
0	0	T4	Natur	Far	Wood	900	0.7151
0	0	T4	Natur	Near	Wood	900	0.7151
1	0	T4	Nehusha	Far	Wood	1500	0.7269
1	0	T4	Nehusha	Near	Wood	1500	0.7269
1	0	T4	Nir_etzion	Far	Wood	950	0.7126
0	0	T4	Nir_etzion	Near	Wood	950	0.7126
1	0	T4	Ofer	Far	Wood	650	0.7731
1	0	T4	Ofer	Near	Wood	650	0.7731
1	0	T4	Paran	Far	Desert	500	0.114
0	0	T4	Paran	Near	Desert	500	0.114
0	0	T4	Sde_boker	Far	Desert	450	0.1345
0	0	T4	Sde_boker	Near	Desert	450	0.1345
0	0	T4	Sekher	Far	Desert	Na	0.2468
0	0	T4	Sekher	Near	Desert	Na	0.2468
1	0	T4	Shaal	Far	Wood	300	0.7405
0	0	T4	Shaal	Near	Wood	300	0.7405
0	0	T4	Shunra	Far	Desert	Na	0.1735
0	0	T4	Shunra	Near	Desert	Na	0.1735
1	0	T4	Yagur	Far	Wood	1650	0.7449
1	0	T4	Yagur	Near	Wood	1650	0.7449
1	0	T4	Yavneel	Far	Wood	4450	0.7152
0	0	T4	Yavneel	Near	Wood	4450	0.7152
1	0	T4	Yeruham	Far	Desert	10,200	0.1384
0	0	T4	Yeruham	Near	Desert	10,200	0.1384
0	0	T4	Yotvata	Far	Desert	700	0.1522
0	0	T4	Yotvata	Near	Desert	700	0.1522
0	0	T4	Zofar	Far	Desert	450	0.1368
1	0	T4	Zofar	Near	Desert	450	0.1368

Campaign: The images T0 were taken in 2013 or 2014, T1 in 2015/16, T2 in 2017/18, and T3 in 2019/20.

Proximity: “near” is typically ~100 m from the last house; “far” is 0.5–2 km from the last house.

Tabby and Non‐tabby: presence or absence of morph in this survey.

NDVI, normalized difference vegetation index (color gradient from yellow = 0 to green = 1).

Population: data from the Bureau of Statistics, mean for the relevant years rounded to the nearest 50.

## APPENDIX 2

### 2.1

Golden jackals (*Canis aureus*) are among the native predators whose detection cats would rather avoid—quite unsuccessfully, in the case of this unfortunate individual.

## References

[ece310261-bib-0001] Allen, W. L. , Cuthill, I. C. , Scott‐Samuel, N. E. , & Baddeley, R. (2011). Why the leopard got its spots: Relating pattern development to ecology in felids. Proceedings of the Royal Society B: Biological Sciences, 278, 1373–1380. 10.1098/rspb.2010.1734 PMC3061134 20961899

[ece310261-bib-0002] Beaumont, M. , Barratt, E. M. , Gottelli, D. , Kitchener, A. C. , Daniels, M. J. , Pritchard, J. K. , & Bruford, M. W. (2001). Genetic diversity and introgression in the Scottish wildcat. Molecular Ecology, 10, 319–336. 11298948 10.1046/j.1365-294x.2001.01196.x

[ece310261-bib-0003] Bengsen, A. J. , Butler, J. A. , & Masters, P. (2012). Applying home‐range and landscape‐use data to design effective feral‐cat control programs. Wildlife Research, 39, 258–265.

[ece310261-bib-0005] Biró, Z. , Lanszki, J. , Szemethy, L. , Heltai, M. , & Randi, E. (2005). Feeding habits of feral domestic cats (*Felis catus*), wild cats (*Felis silvestris*) and their hybrids: Trophic niche overlap among cat groups in Hungary. Journal of Zoology, 266, 187–196.

[ece310261-bib-0006] Bonnaud, E. , Medina, F. M. , Vidal, E. , Nogales, M. , Tershy, B. , Zavaleta, E. , Donlan, C. J. , Keitt, B. , Le Corre, M. , & Horwath, S. V. (2011). The diet of feral cats on islands: A review and a call for more studies. Biological Invasions, 13, 581–603.

[ece310261-bib-0007] Brickner‐Braun, I. , Geffen, E. , & Yom‐Tov, Y. (2007). The domestic cat as a predator of Israeli wildlife. Israel Journal of Ecology and Evolution, 53, 129–142.

[ece310261-bib-0008] Caro, T. (2014). Antipredator deception in terrestrial vertebrates. Current Zoology, 60, 16–25.

[ece310261-bib-0009] Cuthill, I. C. (2019). Camouflage. Journal of Zoology, 308, 75–92.

[ece310261-bib-0010] Daniels, M. J. , Beaumont, M. A. , Johnson, P. J. , Balharry, D. , Macdonald, D. W. , & Barratt, E. (2001). Ecology and genetics of wild‐living cats in the north‐east of Scotland and occurence probability the implications for the conservation of the wildcat. Journal of Applied Ecology, 38, 146–161.

[ece310261-bib-0011] Dards, J. L. , & Robinson, R. (1983). Gene frequencies in a population of feral cats in Portsmouth naval dockyard. Theoretical and Applied Genetics, 64, 197–204. 24264945 10.1007/BF00303764

[ece310261-bib-0012] Driscoll, C. A. , Clutton‐Brock, J. , Kitchener, A. C. , & O'Brien, S. J. (2009). The taming of the cat. Scientific American, 300, 68–75. PMC5790555 19485091

[ece310261-bib-0013] Endler, J. A. (1981). An overview of the relationships between mimicry and crypsis. Biological Journal of the Linnean Society, 16, 25–31.

[ece310261-bib-0014] Farnworth, M. J. , Packer, R. M. , Sordo, L. , Chen, R. , Caney, S. M. , & Gunn‐Moore, D. A. (2018). In the eye of the beholder: Owner preferences for variations in cats' appearances with specific focus on skull morphology. Animals, 8(2), 30. 29461472 10.3390/ani8020030 PMC5836038

[ece310261-bib-0015] Ferreira, J. P. , Leitão, I. , Santos‐Reis, M. , & Revilla, E. (2011). Human‐related factors regulate the spatial ecology of domestic cats in sensitive areas for conservation. PLoS One, 6, e25970. 22043298 10.1371/journal.pone.0025970 PMC3197152

[ece310261-bib-0016] Finkler, H. , Hatna, E. , & Terkel, J. (2011). The influence of neighbourhood socio‐demographic factors on densities of free‐roaming cat populations in an urban ecosystem in Israel. Wildlife Research, 38, 235–243.

[ece310261-bib-0018] Fitzgerald, B. , & Karl, B. (1986). Home range of feral house cats (*Felis catus* L.) in forest of the Orongorongo Valley, Wellington, New Zealand. New Zealand Journal of Ecology, 9, 71–82.

[ece310261-bib-0019] Germain, E. , Benhamou, S. , & Poulle, M. L. (2008). Spatio‐temporal sharing between the European wildcat, the domestic cat and their hybrids. Journal of Zoology, 276, 195–203.

[ece310261-bib-0020] González‐Ramírez, M. T. , & Landero‐Hernández, R. (2022). Cat coat colour, personality traits and the cat‐owner relationship scale: A study with cat owners in Mexico. Animals, 12, 1030. 35454276 10.3390/ani12081030 PMC9024431

[ece310261-bib-0021] Grimm, D. (2009). A cure for euthanasia? Science, 325, 1490–1493. 19762620 10.1126/science.325_1490

[ece310261-bib-0022] Hardman, B. , Moro, D. , & Calver, M. (2016). Direct evidence implicates feral cat predation as the primary cause of failure of a mammal reintroduction programme. Ecological Management & Restoration, 17, 152–158.

[ece310261-bib-0023] Kaelin, C. B. , & Barsh, G. S. (2013). Genetics of pigmentation in dogs and cats. Annual Review of Animal Biosciences, 1, 125–156. 25387014 10.1146/annurev-animal-031412-103659

[ece310261-bib-0024] Konecny, M. J. (1983). Behavioral ecology of feral house cats in the Galapagos Islands, Ecuador (pp. 34–36). University of Florida.

[ece310261-bib-0025] Liberg, O. (1980). Spacing patterns in a population of rural free roaming domestic cats. Oikos, 35, 336–349.

[ece310261-bib-0026] Liberg, O. , & Sandell, M. (2000). Spatial organisation and reproductive tactics in the domestic cat and other felids. In D. C. Turner & P. Bateson (Eds.), The domestic cat: The biology of its behaviour (pp. 83–98). Cambridge University Press.

[ece310261-bib-0027] Lowe, S. , Browne, M. , Boudjelas, S. , & De Poorter, M. (2000). 100 of the world's worst invasive alien species: A selection from the global invasive species database (Vol. 12). Invasive Species Specialist Group.

[ece310261-bib-0028] Lüdecke, D. , Ben‐Shachar, M. S. , Patil, I. , Waggoner, P. , & Makowski, D. (2021). Performance: An R package for assessment, comparison and testing of statistical models. Journal of Open Source Software, 6(60), 3139.

[ece310261-bib-0029] McGregor, H. , Legge, S. , Jones, M. E. , & Johnson, C. N. (2015). Feral cats are better killers in open habitats, revealed by animal‐borne video. PLoS One, 10, e0133915. 26288224 10.1371/journal.pone.0133915 PMC4545751

[ece310261-bib-0030] Mella‐Méndez, I. , Flores‐Peredo, R. , Amaya‐Espinel, J. D. , Bolívar‐Cimé, B. , Mac Swiney, G. M. C. , & Martínez, A. J. (2022). Predation of wildlife by domestic cats in a Neotropical city: A multi‐factor issue. Biological Invasions, 24, 1539–1551.

[ece310261-bib-0031] Mendelssohn, H. , & Yom‐Tov, Y. (1999). Mammalia of Israel. The Israeli Academy of Sciences and Humanities.

[ece310261-bib-0032] Mirmovitch, V. (1995). Spatial organisation of urban feral cats (*Felis catus*) in Jerusalem. Wildlife Research, 22, 299–310.

[ece310261-bib-0033] Montague, M. J. , Li, G. , Gandolfi, B. , Khan, R. , Aken, B. L. , Searle, S. M. , Minx, P. , Hillier, L. W. , Koboldt, D. C. , Davis, B. W. , & Driscoll, C. A. (2014). Comparative analysis of the domestic cat genome reveals genetic signatures underlying feline biology and domestication. Proceedings of the National Academy of Sciences, 111, 17230–17235. 10.1073/pnas.1410083111 PMC4260561 25385592

[ece310261-bib-0034] Murray, D. L. , Boutin, S. , O'Donoghue, M. , & Nams, V. O. (1995). Hunting behaviour of a sympatric felid and canid in relation to vegetative cover. Animal Behaviour, 50, 1203–1210.

[ece310261-bib-0035] Ortolani, A. (1999). Spots, stripes, tail tips and dark eyes: Predicting the function of carnivore colour patterns using the comparative method. Biological Journal of the Linnean Society, 67, 433–476.

[ece310261-bib-0036] Palomares, F. , & Caro, T. M. (1999). Interspecific killing among mammalian carnivores. The American Naturalist, 153, 492–508. 10.1086/303189 29578790

[ece310261-bib-0037] Pierpaoli, M. , Birò, Z. S. , Herrmann, M. , Hupe, K. , Fernandes, M. , Ragni, B. , Szemethy, L. , & Randi, E. (2003). Genetic distinction of wildcat (*Felis silvestris*) populations in Europe, and hybridization with domestic cats in Hungary. Molecular Ecology, 12, 2585–2598. 12969463 10.1046/j.1365-294x.2003.01939.x

[ece310261-bib-0038] Randall, W. , Thomas Cunningham, J. , Randall, S. , Luttschwager, J. , & Johnson, R. F. (1987). A two‐peak circadian system in body temperature and activity in the domestic cat, *Felis catus* L. Journal of Thermal Biology, 12, 27–37.

[ece310261-bib-0039] Randi, E. (2008). Detecting hybridization between wild species and their domesticated relatives. Molecular Ecology, 17, 285–293. 18173502 10.1111/j.1365-294X.2007.03417.x

[ece310261-bib-0040] Sorek, M. , & Shapira, I. (2018). Israel State of Nature Report 2018. Steinhardt Museum of Natural History, Tel Aviv University.

[ece310261-bib-0041] Stelow, E. A. , Bain, M. J. , & Kass, P. H. (2016). The relationship between coat colour and aggressive behaviours in the domestic cat. Journal of Applied Animal Welfare Science, 19, 1–15. 26467020 10.1080/10888705.2015.1081820

[ece310261-bib-0042] Todd, N. B. (1977). Cats and commerce. Scientific American, 237, 100–107.

[ece310261-bib-0043] Wagner, A. , & Wolsan, M. (1987). Pelage mutant allele frequencies in domestic cat populations of Poland. Journal of Heredity, 78, 197–200. 3611716 10.1093/oxfordjournals.jhered.a110356

[ece310261-bib-0044] Yamaguchi, N. , Kitchener, A. C. , Driscoll, C. A. , Ward, J. M. , & Macdonald, D. W. (2004). Craniological differentiation amongst wild‐living cats in Britain and southern Africa: Natural variation or the effects of hybridisation? Animal Conservation, 7, 339–351.

